# Combustible Cigarette and Smokeless Tobacco Product Preparations Differentially Regulate Intracellular Calcium Mobilization in HL60 Cells

**DOI:** 10.1007/s10753-019-01025-x

**Published:** 2019-06-12

**Authors:** S. Arimilli, P. Makena, G.L. Prasad

**Affiliations:** 1 Eurofins Lancaster Laboratories PSS, Winston-Salem, NC 27105 USA; 2 RAI Services Company, Winston-Salem, NC 27105 USA

**Keywords:** combustible, non-combustible, tobacco product preparations, calcium mobilization

## Abstract

Changes in the level of intracellular calcium ([Ca^2+^]_i_) are central to leukocyte signaling and immune response. Although evidence suggests that cigarette smoking affects inflammatory response *via* an increase in intracellular calcium, it remains unclear if the use of smokeless tobacco (*e.g.*, moist snuff) elicits a similar response. In this study, we evaluated the effects of tobacco product preparations (TPPs), including total particulate matter (TPM) from 3R4F reference cigarettes, smokeless tobacco extract (STE) from 2S3 reference moist snuff, and nicotine alone on Ca^2+^ mobilization in HL60 cells. Treatment with TPM, but not STE or nicotine alone, significantly increased [Ca^2+^]_i_ in a concentration-dependent manner in HL60 cells. Moreover, TPM-induced [Ca^2+^]_i_ increase was not related to extracellular Ca^2+^ and did not require the activation of the IP3 pathway nor involved the transient receptor potential (TRP) channels. Our findings indicate that, in cells having either intact or depleted endoplasmic reticulum (ER) Ca^2+^ stores, TPM-mediated [Ca^2+^]_i_ increase involves cytosolic Ca^2+^ pools other than thapsigargin-sensitive ER Ca^2+^ stores. These results, for the first time, demonstrate that TPM triggers [Ca^2+^]_i_ increases, while significantly higher nicotine equivalent doses of STE or nicotine alone, did not affect [Ca^2+^]_i_ under the experimental conditions. In summary, our study suggests that in contrast with STE or nicotine preparations, TPM activates Ca^2+^ signaling pathways in HL60 cells. The differential effect of combustible and non-combustible TPPs on Ca^2+^ mobilization could be a useful *in vitro* endpoint for tobacco product evaluation.

## INTRODUCTION

Chronic smoking is associated with the onset of chronic obstructive pulmonary disease and is a major risk factor for lung cancer and cardiovascular disease [1–5]. Combustible tobacco smoke is a complex mixture of several thousands of chemicals, some of which have been reported to exhibit oxidant, proinflammatory, cytotoxic, mutagenic, carcinogenic, and/or antigenic properties [6–12].

A number of studies have reported that the use of smokeless tobacco (ST) products is associated with a reduced risk of adverse health effects compared with combustible cigarette smoking [13–17]. For cigarette smokers who are unwilling or unable to quit tobacco product use, reducing exposure to potentially harmful cigarette smoke constituents by switching to alternative tobacco products might lower health risks. This concept is the basis of the tobacco harm reduction principle and relies on the existence of a risk continuum across a range of tobacco products, with combustible cigarettes being considered the most harmful tobacco product [14, 18]. We have previously assessed the effects of combustible and non-combustible tobacco product preparations (TPPs) on several functional endpoints in several cell types. For example, we have shown differential effects of combustible and non-combustible TPPs on DNA damage in oral cavity cells [19], regulation of gene expression in cultured human dermal fibroblasts [20], and immune cell function in NK and T cells [21]. Thus, a better understanding of the effects of the tobacco products will aid in developing rapid and functionally relevant tools for tobacco product evaluation.

Chronic smoking impacts several key immune functions, including adaptive and innate immune functions [22–25]. Additionally, smokeless tobacco use is also reported to be associated with immune dysfunction, including the dysregulation of immune cells and their components [26]. Our previous findings have consistently demonstrated that combustible TPPs induce more potent effects on leukocyte functions compared with non-combustible preparations [21, 27].

One of the key molecular events of leukocyte signaling and regulation of immune responses is an increase in intracellular calcium [Ca^2+^]_i_, which, in turn, activates diverse signal transduction pathways involved in regulating cell function. Two major mechanisms contribute to the elevation of [Ca^2+^]_i_ are: (1) store-operated calcium entry (SOCE) from intracellular pools, including the endoplasmic reticulum (ER), mitochondria, and lysosomes and (2) receptor-operated calcium entry (ROCE) of extracellular Ca^2+^. Both processes often occur either simultaneously or sequentially. Additionally, in many excitable cell types, entry of Ca^2+^ through transient receptor potential (TRP) channels can be activated by membrane depolarization [28]. Extracellular calcium mobilization may be triggered by a variety of processes, including direct and indirect activation of cell surface receptors by mechanical, electrical, and chemical stimuli [29–31].

The most common mechanism of voltage-independent Ca^2+^ signaling involves receptor-mediated activation of phospholipase C (PLC). Activated PLC releases inositol 1,4,5-trisphosphate (IP3), which in turn induces the release of Ca^2+^ from intracellular stores (components of the ER) into the cytosol. In non-excitable cells, the most commonly observed mechanism of regulated Ca^2+^ entry is a process known as capacitative Ca^2+^ entry through store-operated Ca^2+^ channels (SOC). *Via* this process, the depletion of intracellular stores, due to the action of IP3 or other Ca^2+^ releasing signals, activates a mechanism that opens plasma membrane Ca^2+^ ion channels known as calcium release-activated channels (CRAC) to replenish ER Ca^2+^ levels [32, 33]. Canonical TRP channels have also been reported to facilitate an increase in intracellular Ca^2+^ concentrations either directly, through coupled plasma membrane receptor stimulation, or through store depletion in different cell types [34–36]. In addition, there are many other non-store-operated routes of Ca^2+^ entry and modulators of lymphocyte cytosolic Ca^2+^ concentration [37–40].

In this study, we evaluated the effects of tobacco product TPM and smokeless tobacco extract (STE) from reference tobacco products as well as nicotine alone, on [Ca^2+^]_i_ in human leukemia HL60 cells. The objective of this study was to evaluate if combustible TPM differentially induces [Ca^2+^]_i_ compared with STE and nicotine alone. We performed a series of experiments to investigate whether (1) [Ca^2+^]_i_ induced by combustible TPM or STE was mediated through SOCE and/or ROCE and (2) the role of IP3 receptors and TRP channels using pharmacological inhibitors to better understand the effect of the TPPs on intracellular Ca^2+^ mobilization.

## MATERIALS AND METHODS

### Cell Culture

HL60 cells were purchased from the American Type Culture Collection (Manassas, VA) and grown in suspension in RPMI complete medium containing L-glutamine and penicillin/streptomycin and supplemented with 10% fetal bovine serum in the presence of 5% CO_2_.

### Tobacco Product Preparations

As described previously [21], total particulate matter (TPM) was prepared by smoking 3R4F reference cigarettes (University of Kentucky) using the standard ISO method (35-mL puff volume, 60-s inter puff interval, and 2-s puff duration) and dissolving the particulate phase in DMSO. Smokeless tobacco extract was prepared by adding 10 g smokeless tobacco reference product, 2S3 (University of Kentucky), into 200 mL 10 mM phosphate buffer solution. The mixture was placed in an incubating shaker set to 37 °C at 250 rpm for 2 h. The STE was centrifuged at room temperature for 15 min at 3000 rpm. Decanted liquid supernatant was filtered through a sterile 1.6-μm glass fiber filter and then filtered through a sterile 0.22-μm cellulose acetate filter. Osmolarity of the final extract was measured and aliquots were stored at ≤ − 70 °C. Neat nicotine (Sigma-Aldrich, Milwaukee, WI) was used as a reference. Aliquots of frozen TPPs were analyzed for nicotine at Labstat International ULC (Kitchener, Ontario, Canada).

### Chemical Analysis

Aliquots of frozen TPM and STE were analyzed to determine the levels of nicotine; final nicotine content of the TPPs was used for calculating the exposure of cells. This allowed for exposures to be conducted on relative nicotine levels or equi-nicotine units. To compare the effects of *in vitro* exposure to TPM and STE, we used equi-nicotine units, expressed in micrograms per milliliter, as a common measure of exposure to the different TPPs [21].

### Fluo-3AM Labeling

In order to measure the changes in [Ca^2+^]_i_ concentration, cells were loaded with Fluo-3AM (Thermo Fisher, Waltham, MA) calcium indicator dye. A Fluo-3AM stock solution of 1.25 mM was prepared in DMSO. Control and treated cells were pelleted and washed in warm MACS running Buffer (Miltenyi Biotec) at a density of 1 × 10^6^ cells/mL. A 4.4 μM working solution of Fluo-3AM was prepared in warm MACS running Buffer and added to the pelleted cells and incubated at 37 °C for 30 min. Cells were washed three times with warm MACS running buffer and resuspended in 400 μL of 1 mM EGTA solution made with MACS running buffer to provide Ca^2+^ free cell suspension. The cell suspensions were then transferred to cluster tubes for acquisition on the flow cytometer (BD Biosciences, San Jose, CA).

### Flow Cytometry Acquisition on BD FACSCalibur and Analysis

Phorbol 12-myristate 13-acetate (PMA), ionomycin, and thapsigargin were purchased from Sigma-Aldrich. PMA stock solution of 1 mg/mL was prepared in DMSO and diluted to a working concentration of 60 ng/mL in RPMI complete media. Ionomycin stock solution of 1 mg/mL was made using DMSO and diluted to a working concentration of 600 ng/ml in RPMI complete medium. A stock solution of 0.5 g/mL (769 mM) thapsigargin was made with DMSO and used at a working concentration of 5 μM. A stock solution of 1 M CaCl_2_ was made in distilled water and used at 0.5 mM working concentration. Ruthenium red (Ru) and SKF-96365 (SK), TRP channel inhibitors, were purchased from Sigma-Aldrich and used at 10 μM working concentration along with the Fluo-3AM labeling as well as during the acquisition. With the cytometer running on low, the fluorescence signals from cell samples were acquired following stimulation at 488 nm wavelength. Basal Fluo-3 fluorescence was determined by an initial measurement at 60 s and subsequently either PMA/ionomycin, or thapsigargin (TG), or the indicated equi-nicotine concentrations of TPPs were added to the cell suspension for additional 4-min acquisitions. TPPs or thapsigargin and CaCl_2_ were then further added, as indicated, and data were acquired for an additional 3 min. As indicated in some experiments, CaCl_2_ was added to the samples at 7 min and the fluorescence signal was recorded for an additional 3 min on the flow cytometer. Data were analyzed offline using FlowJo software (Tree Star, version 9.3.1).

### Area Under the Curve Calculation

The area under the curve (AUC) of all the florescence signals was calculated using the FlowJo software. Using a new workspace, all files from one experiment were selected as samples. Using the kinetics platform, the final kinetic graph was obtained using the mean *Y* value of all the traces selected followed by a Gaussian smoothing. Time slices were then made as follows: 0–60 s, 75–240 s, 255–420 s, and 435–600 s. The AUC was calculated for each time slice after subtraction of the baseline *Y* value (0–60 s time slice) for normalization. All areas belonging to a certain time slice and experimental procedure were then averaged and further analyzed statistically.

### Statistical Analysis

All the results are presented as mean ± standard deviation of the mean AUC (*N* = 3 or 4). Results obtained after TPM and STE exposure were statistically compared with their corresponding controls, DMSO and PB respectively, using the *t* tests. The statistical significance was indicated by *p < 0.05.

## RESULTS

In this study, we evaluated intracellular calcium mobilization in HL60 cells following exposure to different concentrations of TPM, STE, and nicotine alone. We sought to evaluate whether these test materials were able to induce intracellular and/or extracellular calcium mobilization by SOCE and/or ROCE, and quantitatively compare their impacts.

### Effect of TPPs on [Ca^2+^]_i_

**Fig. 1:**
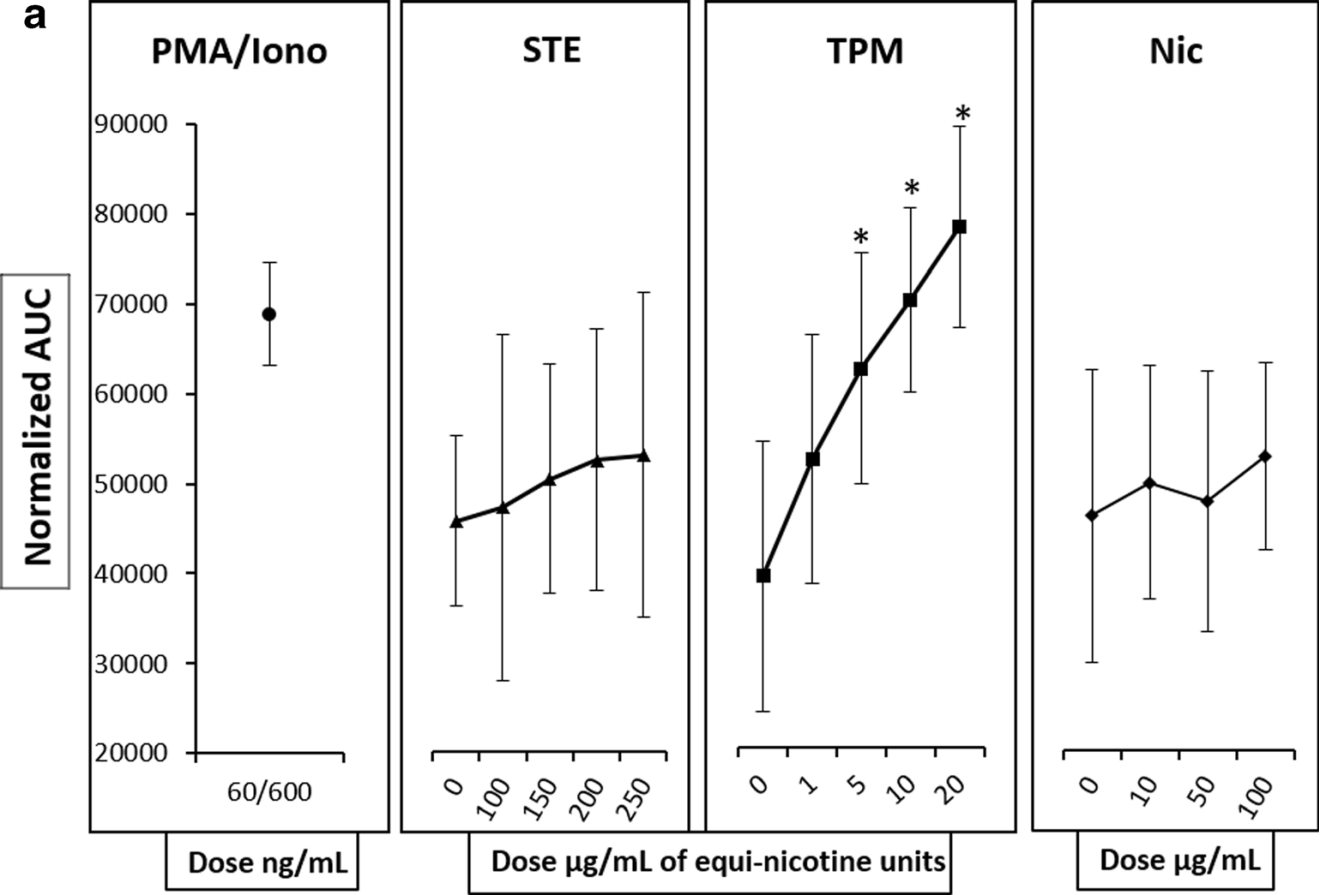

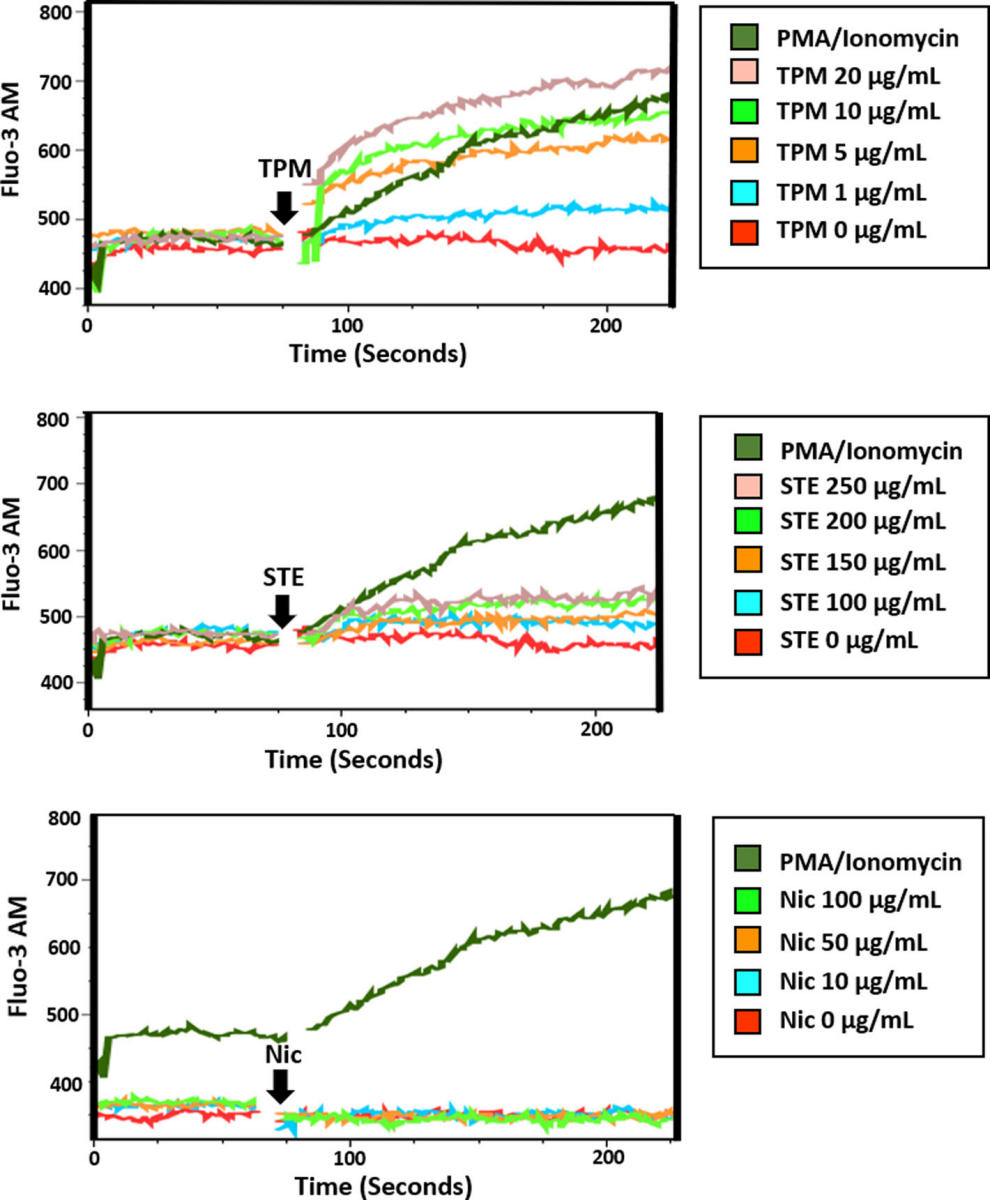
Effect of TPPs on [Ca^2+^]_i_. Fluo-3AM–labeled HL60 cells exposed to equi-nicotine units of TPM and STE or different concentrations of nicotine. Intracellular Ca^2+^ mobilization was measured using flow cytometry. PMA/ionomycin was used as a positive control and DMSO and PB as negative vehicle controls for TPM and STE, respectively. Normalized AUC of PMA/ionomycin and different concentrations of STE, TPM, and nicotine from three independent experiments are shown as mean ± SD (**a**). A linear regression was performed for each treatment to test the dose effect on the [Ca^2+^]_i_; the statistical significance is indicated by *p < 0.05. Flow cytometer raw data of kinetics from HL60 cells exposed to different concentrations of TPM (**b**), STE (**c**), and nicotine (**d**).

In the first set of experiments, we measured [Ca^2+^]_i_ in Fluo-3AM–labeled HL60 cells following exposure to TPM, STE, and nicotine alone. HL60 cells were initially loaded with Fluo-3AM dye and suspended in Ca^2+^ free media. Then, the calcium indicator–loaded cells were exposed to several doses of equi-nicotine units of TPM (1, 5, 10, and 20 μg/mL, Fig. 1a, b), STE (100, 150, 200, and 250 μg/mL, Fig. 1a, c), or nicotine (0, 10, 50, and 100 μg/mL, Fig. 1a, d). The response to PMA and ionomicyn (PMA/Iono), agonists of SOCE and ROCE, respectively, was used as a positive control. DMSO and phosphate buffer (PB) were used as negative controls (0 μg/mL) for TPM and STE treatments, respectively (Fig. 1a–d).

The exposure of HL60 cells to TPM induced a concentration-dependent and statistically significant increase in [Ca^2+^]_i_ (Fig. 1a, b) at all tested concentrations compared with the DMSO vehicle control (*p* < 0.05). In contrast, treatment with STE induced a concentration-dependent, though modest but statistically non-significant, increase in [Ca^2+^]_i_ compared with the PB vehicle control (Fig. 1a). Application of nicotine alone, regardless of the concentrations tested, had no effect on [Ca^2+^]_i_ (Fig. 1a, d). These data suggest that TPM was able to induce [Ca^2+^]_i_ at lower equi-nicotine units in comparison with STE.

### Effect of TPPs on Extracellular Ca^2+^ Influx

**Fig. 2:**
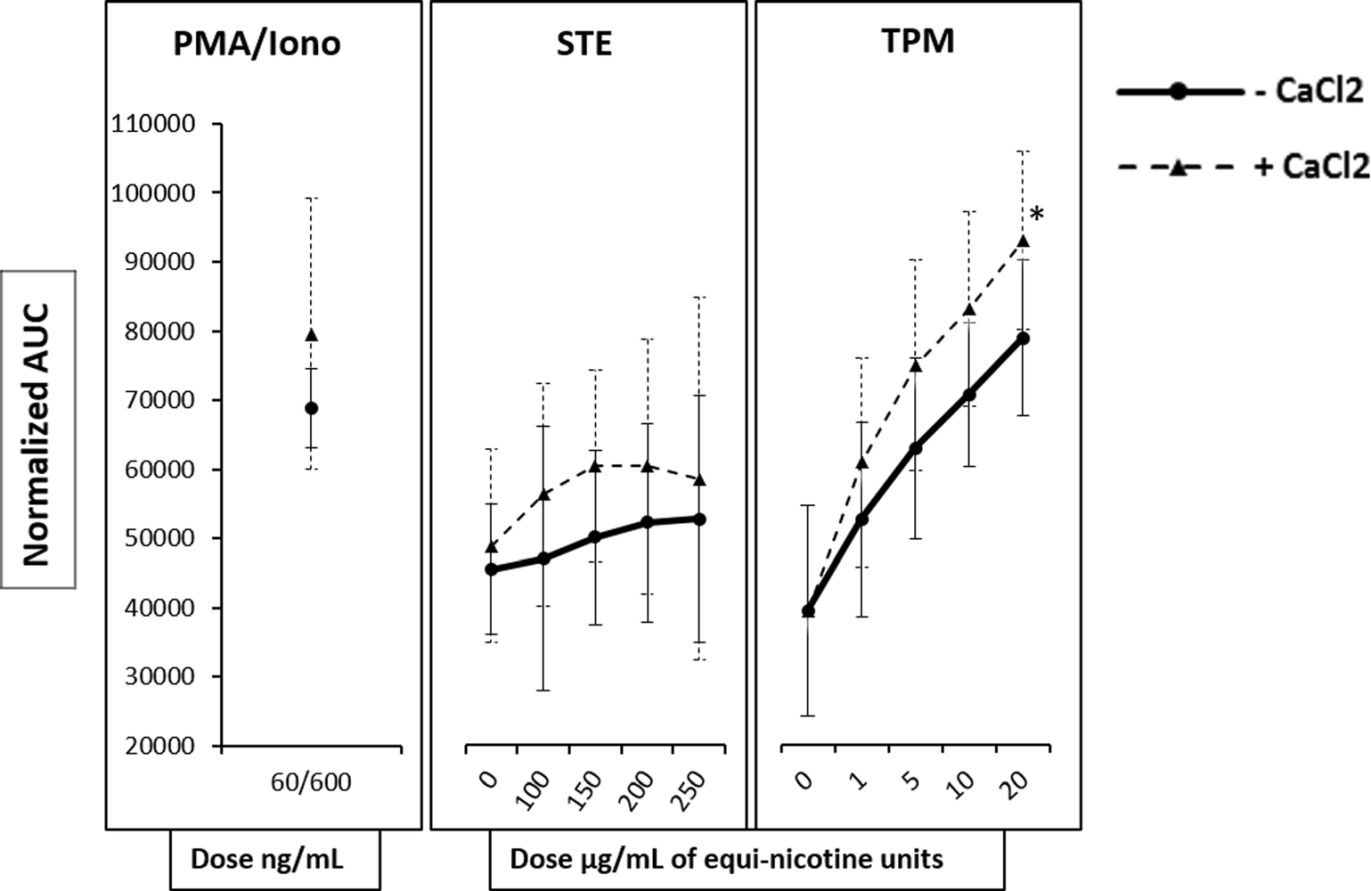
Effect of TPPs on extracellular Ca^2+^ influx. Fluo-3AM–labeled HL60 cells exposed to equi-nicotine units of TPM and STE. a Single concentration of CaCl_2_ was added to each concentration of TPP. Intracellular Ca^2+^ mobilization, [Ca^2+^]_i_, was measured using flow cytometry. DMSO and PB were used as negative vehicle controls for TPM and STE, respectively. Normalized AUC of PMA/ionomycin and different concentrations of TPM and STE from three independent experiments are shown as mean ± SD. For each TPP, two-way analysis of variance and *post hoc* analysis using Tukey’s honestly significant difference test (HSD) was performed.

To assess whether the TPM-induced increase in [Ca^2+^]_i_ is due to extracellular Ca^2+^ influx, we investigated the effect of TPM exposure in cells with intact ER stores and with Ca^2+^ supplemented extracellularly. During the experiment, a single concentration of CaCl_2_ was added to the cells following TPM or STE treatment. Under these experimental conditions, TPM induced a dose-dependent increase in [Ca^2+^]_i_, similar to the [Ca^2+^]_i_ evoked in the absence of extracellular CaCl_2_ (Fig. 2). Notably, the increase in Ca^2+^ flux at a high dose of TPM (20 μg/mL) was significantly larger than the increase at the low 1 μg/mL dose or control. Thus, while the [Ca^2+^]_i_ increase was slightly greater in the presence of extracellular CaCl_2_ at all treatments compared with absence of externally provided calcium, the difference was not statistically significant, indicating that TPM did not mobilize significant extracellular Ca^2+^ when intracellular ER stores were available. In contrast to the effects of CaCl_2_ following TPM exposure, addition of extracellular CaCl_2_ following exposure to different concentrations of STE (Fig. 2) had no significant effect on Ca^2+^ flux.

### Effect of ER Store Depletion on TPP-Induced [Ca^2+^]_i_

In lymphocytes and polymorphonuclear leukocytes, most of the intracellular calcium is stored within the ER. The intracellular calcium concentration is therefore mainly regulated by calcium release from the ER *via* IP3-sensitive receptors, and its subsequent re-uptake into the ER by a calcium pump called sarco(endo)plasmic reticulum calcium ATPase (SERCA). In order to determine the role of ER Ca^2+^ stores on the TPM-induced increase in [Ca^2+^]_i_, we depleted ER Ca^2+^ stores using the SERCA inhibitor TG [41, 42]. We hypothesized that if the TPM- or STE-induced increase in [Ca^2+^]_i_ required ER calcium stores, their depletion by TG would impact the magnitude of the response from the test materials on [Ca^2+^]_i_.

We exposed Fluo-3AM–labeled HL60 cells in Ca^2+^ free media to TG (5 μM), preceding the application of TPM (5, 10, and 20 μg/mL) or STE (100, 200 and 250 μg/mL). To further test whether the TG-induced depletion of intracellular stores could trigger a capacitative Ca^2+^ entry through the plasma membrane, we continued the experiment by adding CaCl_2_ (0.5 mM) to the extracellular media, providing an extracellular calcium source to the cells.

**Fig. 3:**
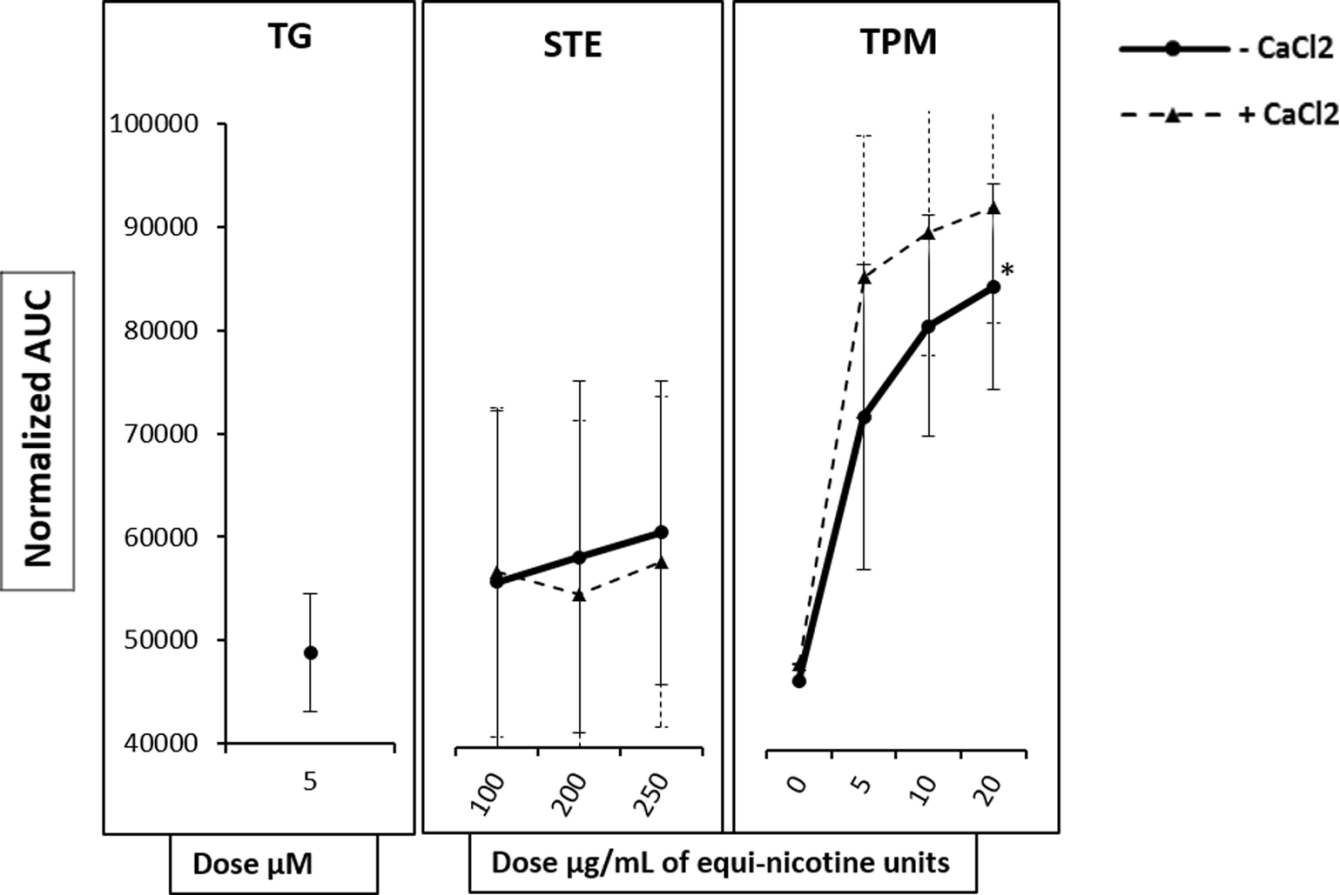
Effect of ER store depletion on TPP-induced [Ca^2+^]_i_. Fluo-3AM–labeled HL60 cells in Ca^2+^ free media were treated with a single concentration of TG before treatment with several equi-nicotine units of TPM (5, 10, and 20 μg/mL) or STE (100, 200, and 250 μg/mL). A single concentration of CaCl_2_ was added to each concentration of TPP. Line graphs are from three independent experiments shown as mean ± SD. For each TPP, two-way analysis of variance was performed to test how [Ca^2+^]_i_ was affected by the presence or absence of CaCl_2_. *Post hoc* analysis using Tukey’s honestly significant difference (HSD) test was performed and no adjusted *p* value was less than 0.05 for the relevant *post hoc* comparison.

Exposure to TPM for 4 min after TG treatment significantly increased [Ca^2+^]_i_ in a dose-dependent manner compared with the DMSO vehicle control (*p* < 0.05) (Fig. 3), indicating that TPM was capable of increasing [Ca^2+^]_i_ independently of ER calcium store release and was potentially mediated by mitochondrial or lysosomal sources. TPM-induced [Ca^2+^]_i_ was further enhanced when CaCl_2_ was added as an extracellular Ca^2+^ source; however, the difference was not statistically significant. TG treatment had no effect following STE exposure in the absence or presence of extracellular calcium.

### Effects of TRP Channel Inhibition on TPM-Induced Calcium Influx

**Fig. 4:**
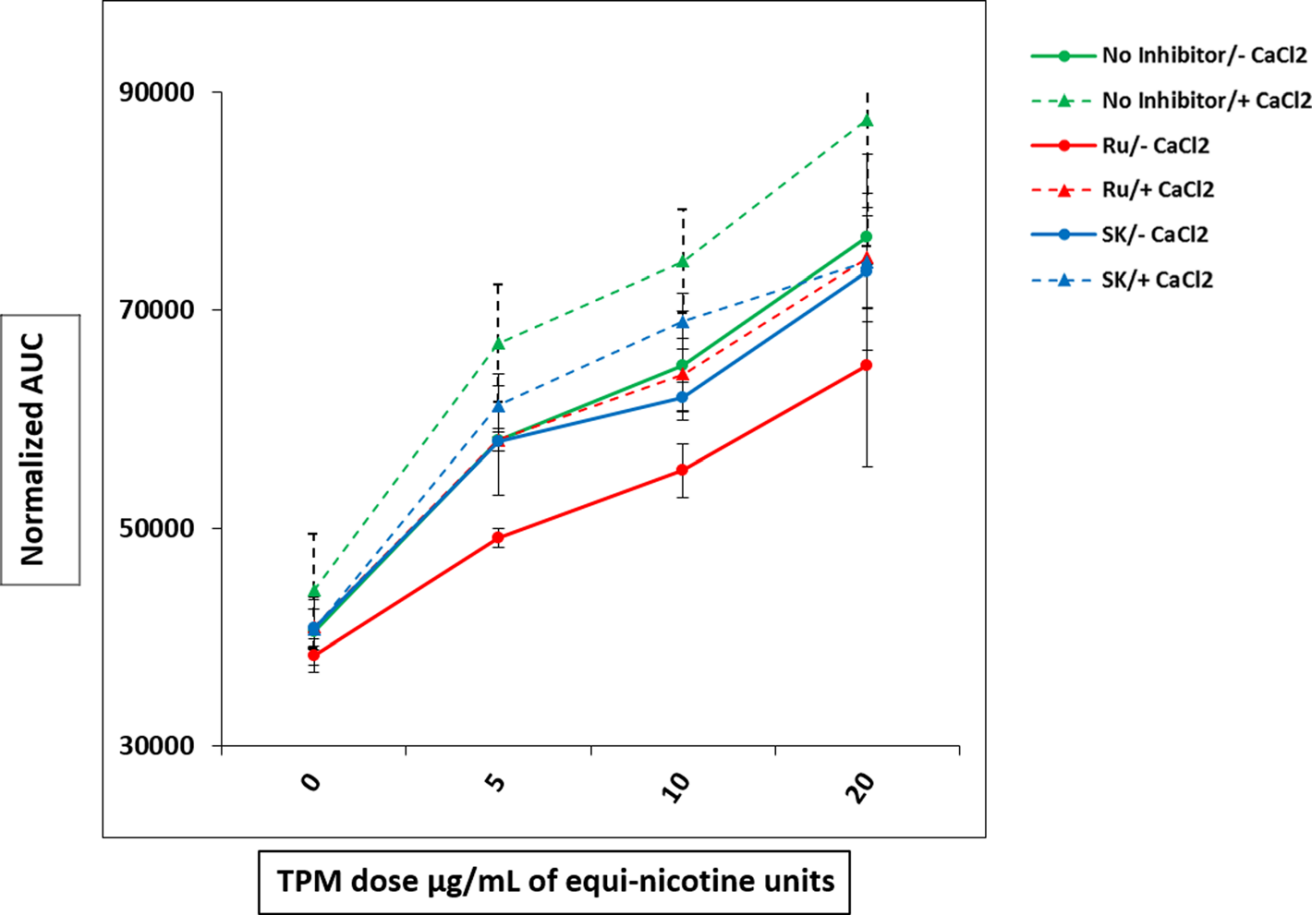
Effects of TRP channel inhibitors on TPM-induced calcium influx. Fluo-3AM–labeled cells were grown in Ca^2+^ free media with TRP channel inhibitor ruthenium red (Ru) or SKF-96365 (SK) and then treated with three concentrations of TPM (5, 10, and 20 μg/mL) followed by a single concentration of CaCl_2_. DMSO was used as a negative vehicle control. Line graphs are from four independent experiments shown as mean ± SD.

To further investigate the mechanisms underlying TPM-induced [Ca^2+^]_i_ increase, we tested the role of TRP channels present on the plasma membrane. HL60 cells were maintained in Ca^2+^-free media together with non-selective TRP channel blockers, either ruthenium red (Ru; 10 μM) or SKF-96365 (SK; 10 μM) (Fig. 4). The Fluo-3AM–labeled cells were then exposed to three different concentrations of TPM (5, 10, and 20 μg/mL) followed by a single concentration of CaCl_2_ (0.5 mM). DMSO was used as a negative vehicle control.

When TRP channels were blocked by Ru or SK pretreatment, TPM-induced [Ca^2+^]_i_ in the presence of extracellular CaCl_2_ tended to be lower at all TPM concentrations, compared with the response with no inhibitors. However, the differences were not statistically significant. Ru and SK had no effect on the DMSO vehicle control group (Fig. 4). In the absence of extracellular calcium, the blocker Ru also seemed to decrease TPM (5 and 10 μg/mL)-induced [Ca^2+^]_i_ increase, suggesting that an intracellular, Ru-sensitive pathway could be involved. However, the difference was not statistically significant compared to the response with no inhibitors. Altogether, these results indicate that TRP channels do not play a major role in calcium influx mechanisms responsible for the TPM-induced [Ca^2+^]_i_ increase.

## DISCUSSION

Several adverse cellular effects of combustible TPPs, in particular TPM, are reported to be associated with increased cytotoxic [27, 43–45], genotoxic [44], and proinflammatory effects [20]. However, the mechanism underlying TPP-induced [Ca^2+^]_i_ mobilization from internal and/or external Ca^2+^ sources remains unclear. To understand the complex response to TPPs that directly and/or indirectly affects cellular functions, we assessed the effect of TPM and STE on [Ca^2+^]_i_ in an established *in vitro* model using human promyelocytic leukemia cells, the HL60 cell line.

In this study, we sought to elucidate the pathways mediating any TPP-induced [Ca^2+^]_i_ increase. This allowed us to compare data obtained using different TPP sources (combustible cigarettes or smokeless tobacco), with a view to understanding if combustible and smokeless tobacco preparations demonstrate different impacts on intracellular calcium levels and, possibly, the underlying mechanisms of action. A key finding of the present study shows that TPM treatment increased [Ca^2+^]_i_ in a dose-dependent manner, starting at very low equi-nicotine concentrations.

Other studies have similarly reported that cigarette smoke extract triggers [Ca^2+^]_i_ mobilization [46–49]. In contrast to the findings with TPM, we demonstrated for the first time that a smokeless TPP, STE, did not trigger similar [Ca^2+^]_i_ increases, even at equi-nicotine concentrations that are two orders of magnitude higher than the TPM tested in the same cell type used in this study. For example, TPM-mediated [Ca^2+^]_i_ increase occurred at equi-nicotine concentrations as low as 1 μg/mL, whereas a higher equi-nicotine level of 250 μg/mL of STE had no effect. These results suggest that combustion-related toxicants may play a role in the mobilization of [Ca^2+^]_i_ when compared with smokeless tobacco products in the HL60 leukemia cell line. In line with our findings, Sassano et al. (2017) assessed the impact of 13 individual tobacco smoke constituents on [Ca^2+^]_i_ in HEK293T cells and found that 1-aminonaphthalene, formaldehyde, and nicotine-derived nitrosamine ketone (NNK) were able to elicit [Ca^2+^]_i_ increases [48].

In our study, testing nicotine alone, even at concentrations two orders of magnitude higher (100 μg/mL) than those present in the TPM, did not induce changes in [Ca^2+^]_i_. This finding is in apparent contrast with the findings of Sassano et al. (2017) who found that nicotine triggered increases in [Ca^2+^]_i_ in both HEK293T and human bronchial epithelial cells (HBEC). However, their study did not compare equi-nicotine units of nicotine and TPPs. Hence, the relative impact of nicotine merits further characterization using different cell types.

To elucidate the pathways involved in TPM-induced increase in [Ca^2+^]_i_ in the HL60 cell line, we evaluated capacitative Ca^2+^ mobilization by supplementation of external CaCl_2_. Our results indicated that TPM-induced mobilization of extracellular CaCl_2_ is not significant. Similarly, Rasmussen et al. (2014) reported that the vapor phase of cigarette smoke increased [Ca^2+^]_i_ and that this increase was unaffected by extracellular Ca^2+^ chelation [47]. Our results, based on the inhibition of the TRP channel, also indicated that TRP channels did not play a major role in TPM-induced calcium influx.

We then intended to identify the intracellular source of TPM-induced Ca^2+^ response by TPM exposure before, or after, depleting ER Ca^2+^ stores by TG. In both scenarios, TPM triggered [Ca^2+^]_i_ increase independently of ER store depletion, suggesting that TPM-mediated [Ca^2+^]_i_ increase occurred through TG-insensitive Ca^2+^ stores. This finding is in line with published work from Rasmussen et al. [47] showing that cigarette smoke neither activates nor inhibits ER Ca^2+^ release. Rasmussen et al. reported that cigarette smoke triggered a rise in cytoplasmic Ca^2+^ in HBECs, BHK^CFTR^, HEK293T, and CALU3 cells that may have emanated from lysosomes [47].

Cytosolic Ca^2+^ is a key second messenger that regulates several functions including cell growth/cell division, apoptosis, and the secretion of both ions and macromolecules [48, 50–53]. Calcium signals in cells of the immune system regulate cell differentiation, gene transcription, and effector functions crucial for the physiological functioning of the immune responses.

To our knowledge, this is the first study to assess and compare the effects of combustible and non-combustible tobacco product exposures on cellular Ca^2+^ mobilization. Our results suggest that TPM-induced Ca^2+^ mobilization may activate Ca^2+^-dependent signaling pathways, potentially leading to altered inflammatory immune responses in HL60 cells compared with smokeless/non-combustible tobacco products. Further work will be necessary to understand the molecular mechanisms underlying these biological effects.

## CONCLUSION

In this study, we evaluated the effects of TPPs from combustible and non-combustible tobacco products, as well as of nicotine alone, on cellular Ca^2+^ mobilization in HL60 cells. We found that TPM, but not STE or nicotine, induced a significant increase in [Ca^2+^]_i_ and could therefore activate Ca^2+^-dependent signaling pathways, potentially leading to altered inflammatory responses in leukocytes. This study provides an understanding of the differential effects of different tobacco products on inflammatory responses in HL60 cells. Additional studies are warranted to identify the source of TPM-induced intracellular calcium mobilization.

## Abbreviations

AUC: area under the curve
COPD: chronic obstructive pulmonary disease
CRAC: calcium release-activated channel
CS: cigarette smoke
CVD: cardiovascular disease
ER: endoplasmic reticulum
IP3: inositol 1,4,5-trisphosphate
nAChR: nicotinic acetylcholine receptors
Nic: nicotine
ORAI1: calcium release-activated calcium channel protein 1
PAH: polycyclic aromatic hydrocarbon
PB: phosphate buffer
PMA: phorbol 12-myristate 13-acetate
PLC: phospholipase C
ROCE: receptor-operated calcium entry
RU: ruthenium red
SERCA: sarco(endo)plasmic reticulum calcium-dependent ATPase
SK: SKF-96365
SOCE: store-operated calcium entry
ST: smokeless tobacco
STE: smokeless tobacco extract
STIM1: stromal interaction molecule 1
TG: thapsigargin
TPM: total particulate matter
TPP: tobacco product preparation
TRP: transient receptor potential
